# Biopolymer-Based Nanoparticles for Drug/Gene Delivery and Tissue Engineering

**DOI:** 10.3390/ijms14011629

**Published:** 2013-01-14

**Authors:** Sachiko Kaihara Nitta, Keiji Numata

**Affiliations:** Enzyme Research Team, RIKEN Biomass Engineering Program, RIKEN, Saitama 351-0198, Japan; E-Mail: sachiko_kaihara@hotmail.com

**Keywords:** biopolymer, nanoparticle, drug delivery, gene delivery, biodegradable polymer

## Abstract

There has been a great interest in application of nanoparticles as biomaterials for delivery of therapeutic molecules such as drugs and genes, and for tissue engineering. In particular, biopolymers are suitable materials as nanoparticles for clinical application due to their versatile traits, including biocompatibility, biodegradability and low immunogenicity. Biopolymers are polymers that are produced from living organisms, which are classified in three groups: polysaccharides, proteins and nucleic acids. It is important to control particle size, charge, morphology of surface and release rate of loaded molecules to use biopolymer-based nanoparticles as drug/gene delivery carriers. To obtain a nano-carrier for therapeutic purposes, a variety of materials and preparation process has been attempted. This review focuses on fabrication of biocompatible nanoparticles consisting of biopolymers such as protein (silk, collagen, gelatin, β-casein, zein and albumin), protein-mimicked polypeptides and polysaccharides (chitosan, alginate, pullulan, starch and heparin). The effects of the nature of the materials and the fabrication process on the characteristics of the nanoparticles are described. In addition, their application as delivery carriers of therapeutic drugs and genes and biomaterials for tissue engineering are also reviewed.

## 1. Introduction

### 1.1. Biopolymers for Therapeutic Application in Nanotechnology

Nanotechnology has been attending much attention since 1980s and has been adapted into many engineering fields such as electronics, mechanical, biomedical and space engineering. In particular, nanotechnology has led to the significant progress in a biomedical field such as controlled drug/gene delivery [1,2], tissue engineering [3,4], imaging of specific sites and probing of DNA structure [5–7]. Among nanomaterials, nanoparticles have been contributing to the progress in this field. In particular, therapies using nanoparticles have widely been achieved for the treatments of cancer [8], diabetes [9], allergy [10], infection [11] and inflammation [12].

The reasons that nanoparticles have been used in therapeutic application are the fact that nanoparticles exist in the same size domain as proteins. Their large surface areas can also allow for displaying a large number of surface functional groups such as ligands. Furthermore, they have a rapid absorption and release behavior provided by high abilities of their diffusion and volume change. In addition, the particle sizes and surface characteristics of nanoparticles can be tailored or controlled. Examples of surface modification of nanoparticles are covalent binding between surface and functional molecules or polymers and layer-by-layer assembly [13]. While organic, inorganic or organic/inorganic hybrid materials are used for the fabrication of nanoparticles, polymeric nanoparticles have also been utilized in therapeutic application. In particular, they are often used as biomaterials for delivery carriers of therapeutic molecules such as drugs and genes [1,2] and for tissue engineering scaffolds [3,4]. Although polymeric nanoparticles have the difficulty of scaling up, low drug-loading capacity and wide size distribution, they have attracted increasing attention from chemists, biologists, engineers and pharmaceutical scientists because they provide the possibility of transporting bioactive compounds to specific tissues, cells and cell compartments [14,15]. Compared to ceramic or metal nanoparticles, polymeric nanoparticles can be fabricated in a wide range of sizes and varieties and can sustain localized drug therapeutic agent for weeks [16]. Whereas both naturally derived and synthetic biomaterials have advantageous features, naturally derived biomaterials have merits of biocompatibility, biodegradability and low immunogenicity. Thus, in this review, we focus on the utilization of biopolymers such as proteins, polypeptides and polysaccharides as biomaterials for drug/gene delivery as well as tissue engineering application.

Generally, the well-defined structure of synthetic polymers exhibit well-defined and fine-tunable degradation kinetic as well as mechanical properties [17]. In comparison, proteins offer several advantages over synthetic polymers as they are metabolizable by digestive enzymes into innocuous peptides whereas synthetic polymers may accumulate in the body above a certain molecular weight and result in toxic degradation products [18]. They also exhibit several drug loading mechanisms including electrostatic attractions, hydrophobic interactions and covalent bonding. Moreover, protein-based nanoparticles offer various possibilities for surface modification due to the presence of functional groups on the surface of the nanoparticles thus enabling specific drug targeting to the site of action. Similarly to proteins, polysaccharides are also digested by the specific enzyme [19]. As far as prolonging circulation time, polysaccharides have advantages over the synthetic polymers such as poly(ethylene glycol) [20].

### 1.2. Nanoparticles for Drug/Gene Delivery

In recent years, a number of polymeric drug/gene-loaded nanoparticles have been developed as drug delivery carriers and their mechanism of circulation in human bodies has been extensively investigated [21,22]. When drugs- or genes-loaded nanoparticles are injected into bodies, they cross epithelial barriers and circulate in the blood vessels before reaching the target site. Escape of nanoparticles from the vascular circulation then occurs in either continuous or fenestrated tissues. At continuous vascular endothelium in healthy tissues, nanoparticles escape from the bloodstream via paracellular pathway, intracellular process or transmembrane transport. At fenestrated vascular endothelium in pathological tissues, on the contrary, the gaps of the fenestration sites on the endothelium are much larger (100 nm to 2 μm) than in healthy tissues (2–6 nm). Therefore, nanoparticles go through fenestrations thus enhancing drug penetration in tissues, and accumulating drugs in tumor sites which is called “enhanced permeation and retention effect (EPR effect)” [22–24]. It should be noted that tumor vasculature fenestrations vary depending on a myriad of factors including cancer type, stage of disease and site in the body. In addition, fenestrations and the vasculature can undergo modifications under various pathological conditions [23,25]. For instance, tumor growth induces the development of neovasculature characterized by discontinuous endothelium with large fenestrations of 200–780 nm allowing nanoparticles passage [26].

There also needs to be toxicology on the particles since they contain various interactions of nanoparticles with fluids, cells, and tissues need to be considered, starting at the portal of entry and then via a range of possible pathways towards target organs [20]. At the site of final retention in the target organ(s), nanoparticles may trigger mediators which then may activate inflammatory or immunological responses. With such reasons, designing the biopolymer-based nanoparticles with the specific sizes is one of the most important criteria for delivery carrier application [27,28]. Particle shape, surface charge and the surface feature also play roles in intercellular delivery since they all affect the mechanism of cellular internalization via endocytosis [19,29]. In addition, to achieve the site-specific delivery and release of bioactive drugs at required rate and quantity, the type of polymers, particle sizes, solubility, biodegradability and surface properties need to be considered [30]. The introduction of stimuli-sensitivity to the carriers is critical for drug delivery system (DDS) application to achieve the controlled release of drugs from the nanoparticles after accumulation at a specific site. By responding to stimuli such as temperature, pH or ionic strengths, the carriers may degrade or diffuse to release the encapsulated drugs. In particular, the addition of pH-sensitivity to the nanoparticles is effective for the DDS application, because of the difference of extracellular pH of normal tissue (pH 7.2–7.4) and many solid tumors (pH 6.2–6.9).

Gene therapy has been applied in many different diseases such as cancer, AIDS, and cardiovascular diseases, and is based on the concept that human disease may be treated by the transfer of genetic materials into specific cells of a patient to supply defective genes responsible for disease development [31]. To transfer the genes to the specific site, genes must escape the processes that affect the disposition of macromolecules. Furthermore, the degradation of gene by serum nucleases needs to be avoided. Thus, encapsulation of genes in delivery carrier is necessary to protect the gene until it reaches its target. The delivery carries must be small enough to internalize into cells and passage to the nucleus. They also need to be capable of escaping endosome–lysosome processing and following endocytosis [31]. While both viral and non-viral vectors have been developed for the delivery of genes, non-viral vectors have been studied more actively due to their low immunogenicity and ease of control their properties [32,33]. Thus, the cationic polymers have a potential for DNA complexation as non-viral vectors for gene therapy applications. To introduce the specificity into the nanoparticle surfaces, the conjugation of cell-specific ligands to the surface of nanoparticles allows for targeted transgene expression. For example, the nanoparticles of genes and the cationic polymers can be modified with proteins (knob, transferrin or antibodies/antigens) to allow for cell-specific targeting and enhanced gene transfer [34].

Nitric oxide (NO)-releasing materials has also emerged as potential therapeutics that exploit NO’s vast biological roles. Nitric oxide is known to be involved in wound healing and to have antimicrobial actions [35]. Thus, studies on various regulatory, protective and deleterious effects of nitric oxide (NO) have prompted intense research activity in the design and synthesis of NO-donating drugs and materials. NO-releasing scaffolds including nanoparticles are particularly promising due to their ability to store and deliver NO payloads in a more controlled and effective manner [36]. In fact, various NO-donating compounds have been incorporated or immobilized in biocompatible polymer matrices and such materials have been used as patches, wound dressings, coatings on blood-contacting medical devices and time-release NO drugs [37]. Biopolymers such as chitosan and dextran have been used as matrices of controlled release of NO [38].

### 1.3. Nanoparticles for Tissue Engineering

Tissue engineering can be considered as a special case of drug delivery where the goal is to accomplish controlled delivery of cells. Controlled release of therapeutic factors enhances the efficacy of tissue engineering. The biological functions of encapsulated drugs and cells can be dramatically enhanced by designing biomaterials with controlled organizations at the nanometer scale [39]. The incorporation of gene delivery elements into the scaffold has great potential to enhance the interplay between cells and the extracellular milieu since delivery of genes to the specific sites introduces signals and cues to cells in a spatial and temporal manner for tissue growth and maintenance. Thus, the therapeutic genes can enhance incorporation of a tissue construct, growth and assimilation with neighboring tissues. Moreover, the delivery of genes using biopolymers can function as not only DNA complexing agents but also structural scaffolds for tissue engineering application. This combination of gene therapy and tissue engineering within a single system is thought to be a new treatment for regeneration medicine [34]. Local gene delivery system using gene-activated matrix (GAM) blends these two strategies, serving as a local bioreactor with therapeutic gene expression and providing a structural template to fill the lesion defects for cell adhesion, proliferation and synthesis of extracellular matrix [40].

## 2. Protein/Polypeptide Nanoparticles

### 2.1. Protein-Based Nanoparticles

Proteins are naturally-derived polymers that are advantageous in their biodegradability, low toxicity, nonantigenicity, high nutritional value, high stability and binding capacity of various drugs such as paclitaxel and ibuprofen [41–44]. Interestingly, they have abilities of emulsification, gelation, forming and water binding capacity [45–47]. Because of these unique properties that are different from any synthetic polymers, protein-based nanocarriers are promising candidates for drug and gene delivery. Naturally derived proteins such as collagen [48,49], elastin [50] and fibronectin [51] have originally been used as biomaterials. Recently, genetically engineered proteins and polypeptides have been produced to manipulate the properties of the biomaterials such as degradation rate, biocompatibility and cell penetration ability by generating new protein sequences, new self-assembling peptides or fusion of different bioactive domains or protein motifs. Elastin-like polypeptides (ELP) are the most commonly studied genetically engineered protein, which will be introduced in Section 2.7 [52–54].

Preparation of nanoparticles from proteins enables to obtain precisely formed nanoparticles since the molecular sizes of proteins are determined by their secondary structures. A variety of proteins such as silk [55,56], albumin [47,57,58], collagen [59,60] and elastin [50] are good examples of proteins that have been utilized for the preparation of therapeutic nanoparticles. Besides protein-based nanoparticles, nanoparticles are also prepared from polypeptides (MW < 10,000) that mimic naturally derived proteins, which contain the features of proteins that form α-helix, β-sheet or random structures are determined by the types of amino acids and the molecular weights of their units. Consequently, they are easily self-assembled to form not only particles but also fibers, sheets, *etc.*

### 2.2. Silk-Based Nanoparticles

Silk proteins are promising materials as biomaterials due to their slow biodegradability, biocompatibility, self-assembling property, excellent mechanical property (tensile strength and Young’s modulus) and controllable structure and morphology [18,61]. Silk proteins have been produced by spiders and insects such as silkworms, and they form fibrous materials. Recombinant silks are also synthesized by the elucidation of silk genetics, structures and biophysics.

Silk-based nanoparticles are often produced by silk fibroins. Stable, spherical, negatively charged and low toxic silk nanoparticles (150–170 nm) have been prepared from silk fibroin solutions of domesticated *Bombyx mori* and tropical tasar silkworm *Antheraea mylitta* [62]. These nanoparticles were accumulated in the cytosol of murine squamous cell carcinoma cells and showed a significantly sustained release of loaded growth factor over 3 weeks. Not only single silk particles, silk-based complexes have also been prepared by the conjugation with other polymers. Silk fibroin and chitosan polymers were blended noncovalently to form nanoparticles (<100 nm) for local and sustained therapeutic curcumin delivery to cancer cells [63]. Conjugation of bioactive agents such as drugs or peptides into silk-based nanoparticles is also efficient methodology for delivery of these molecules into target sites. The crystalline silk protein nanoparticles (40–120 nm) have been conjugated with insulin via covalent crosslinking [64]. It was confirmed that *in vitro* stability of insulin in human serum was enhanced and the half-life of insulin prolonged by conjugating with silk protein nanoparticles. Silk fibroin was also bioconjugated with L-asparaginase to form crystalline nanoparticles with 50–120 nm in diameter [65]. These bioconjugates had a greatly increased resistance to trypsin digestion, and better stability in serum, storage stability in solution, and no leakage of the enzyme from the nanoparticles. To control the release rate of the loaded bioactive molecules in silk-based nanoparticles, dual-drug release system based on silk nanoparticles and the molecular networks of silk hydrogels has been developed [66]. Model drugs incorporated in the silk nanoparticles and silk hydrogels showed fast and constant release, respectively, indicating successful dual-drug release from silk hydrogel containing silk nanoparticles. Further, the nanoparticles composed of DNA and recombinant silks, which contained cell-penetrating peptide, tumor-homing peptide, Arg-Gly-Asp (RGD) motifs and/or cationic sequences, have been designed for gene therapy [66–70].

### 2.3. Collagen- and Gelatin-Based Nanoparticles

Collagen is the main component of extracellular matrix and has been widely used as biomaterials for years due to their promising biocompatibility, low antigenicity and biodegradability [71]. Although collagen forms hydrogels without the use of chemical crosslinking, nanoparticle preparation needs additional chemical treatments due to their weak mechanical strengths. For instance, collagen nanoparticles are often prepared by electrostatic interactions with sodium sulfate employed as a desolvating agent [72]. Recent study demonstrated the preparation of collagen-based nanoparticles (340 nm) with methods of using lipid vesicle cages which allow controlling both the particles dimension and the gelling environment during the collagen polymerization [59]. Due to their ease to control their particle sizes, a large surface area, high adsorption capacity and dispersion ability in water, collagen nanoparticles exhibited sustained releasing of various drugs.

Gelatin is obtained from collagen by acid and alkaline hydrolysis consisting of glycine, proline and 4-hydroxyproline residues with typical structure of -Ala-Gly-Pro-Arg-Gly-Glu-4Hyp-Gly-Pro. Gelatin solution undergoes coil-helix transition followed by aggregation of the helices by the formation of collagen-like triple-helix, enabling the formation of nanoparticles. Moreover, their high number of functional groups on polymer backbone can be used for chemical modification such as crosslinking and addition of ligands. Thus, gelatin is a biopolymer for the production of nanoparticles as delivery carriers.

A number of methods has been reported to prepare gelatin-based nanoparticles including desolvation [73,74], a thermodynamically driven self-assembly process for polymeric materials, emulsion [75], crosslinking with polyethylenimine [76] and glutaraldehyde [77], nanoprecipitation [78], coacervation [79], grafting of hydrophobic anhydrides to the amino groups of primitive gelatin to form self-assembled micelles [80].

Recent studies have shown the availability of the loading of therapeutic molecules into gelatin-based nanoparticles. Insulin-loaded gelatin nanoparticles were prepared for diabetes therapy by a novel water-in-water emulsion technique with gelation by glyceraldehyde. Blood glucose level curves showed obvious decreases in the first 4 h after intratracheal stillation in rats indicating their fast and stable hypoglycemic effect [75]. A photodynamic agent, Hypocrelin b was also loaded in poly(ethylene glycol) modified gelation nanoparticles for photodynamic cancer therapy. The nanoparticles were taken in tumor cells resulting in significant regression of solid tumors [81]. Ciplatin-loaded gelatin nanoparticles with surface-functionalization by activated heparin were developed for the breast cancer therapy. It was indicated that heparin-functionalized nanoparticles had greater uptake to human breast cancer cells than non-treated nanoparticles [82]. Paclitaxel-loaded gelatin nanoparticles were also prepared to intravesical bladder cancer therapy [83]. They showed low intravesical absorption, favorable bladder tissue/tumor targeting and retention behavior for at least 1 week.

### 2.4. β-Casein-Based Nanoparticles

β-Casein is the major milk protein component and is easily self-assemble into micellar structure by intermolecular hydrophobic interactions due to its amphiphilic nature, which is a suitable feature for the application as delivery carriers [84]. The spherical casein micelle has a hydrophobic interior, surrounded by a hydrophilic κ-casein layer that stabilizes the micelle through steric and electrostatic effects [46]. 15–60 β-casein molecules form β-casein micelles with the radius of 7–14 nm [85]. Changes in temperature, pH, ionic strength, water activity and high hydrostatic pressure treatment lead to changes in size distribution of casein micelles because of the absence of a rigid three dimensional tertiary structure.

To utilize β-casein micelles for delivery carriers, it is critical to stabilize the micelles by crosslinking. Crosslinking of lysine residues in casein by glutamine residues of transglutaminase (TGase) increased the intra-micellar stability of casein micelles [86,87]. Chemical crosslinkers such as 1-ethyl-3-(3-dimethylaminopropyl) carbodiimide has also been used to create thermally responsive nanoparticles from β-casein [88]. To further stabilize the casein nanoparticles, the self-assembly of β-casein and lysozyme, followed by heat gelation of lysozyme to entrap casein in the gel were used. The obtained nanoparticles had a spherical shape and their sizes depended on the pH of the heat treatment (100 nm and 300 nm at pH 10.0 and 5.0, respectively) and the molar ratio of β-casein to lysozyme [89].

Since β-casein is an edible material, it is often used as a drug carrier for an oral-delivery system. Several types of hydrophobic chemotherapeutics such as mitoxantrone, vinblastine, irinotecan, docetaxel and paclitaxel have been entrapped in β-casein micelles for target-activated release of drugs for oral delivery application [90]. With digestion of casein with pepsin, paclitaxel retained its cytotoxic activity to human N-87 gastric cancer cells, whereas β-casein-paclitaxel nanoparticles were non-cytotoxic without prior simulated gastric digestion [90]. The gastric digestibility of β-casein can lead to their application for targeting stomach tumors [91].

### 2.5. Zein-Based Nanoparticles

Zein is a water-insoluble but alcohol-soluble protein that is stored in corn kernels. Due to its biocompatibility, low water uptake value, high thermal resistance and good mechanical property, zein has been used as edible coating for foods and pharmaceuticals [92]. Moreover, because zein consists of both hydrophobic and hydrophilic amino acid residues and exists as small globules with diameters between 150 and 550 nm, it has been applied as a promising carrier for encapsulation and controlled release of hydrophobic compounds [93].

Zein nanoparticles have been produced by many methodology including liquid-liquid dispersion process [93,94], phase separation system [92] and nanoprecipitation [95]. Zein nanoparticles (100–200 nm) were formed via a liquid–liquid dispersion process using ethanol and deionized water as an alternative to emulsions. This process is advantageous over conventional emulsification methods in its ease to reduce the particle size and increase the scalability [93,94]. Zein nanoparticles have also been prepared by phase separation process to encapsulate 5-fluorouracil that target liver through intravenous delivery [92,96]. *In vivo* study showed that the particles were mostly accumulated in liver and adequately remained in blood for at least 24 h due to tis relatively higher molecular weight and smaller particle size. PH-controlled nanoprecipitation method was demonstrated to obtain zein nanoparticles with a mean particle size of 365 nm using lecithin and pluronic F68 as stabilizers [95]. The release of 6,7-dihydroxycoumarin from zein nanoparticles was sustained in phosphate buffer (pH 7.4) for up to 9 days. To enhance drug entrapment efficiency, not only solid but hollow zein nanoparticles (60–130 nm) have been developed by introducing sodium carbonate as a template [97]. Hollow nanoparticles are advantageous in their capacity to load a large amount of drug and ability to penetrate into the cell cytoplasm. Metformin in hollow zein nanoparticles showed a more sustained and controlled release profile than that in solid zein nanoparticles. Moreover, hollow zein nanoparticles were found to be able to enter the fibroblast cells 1 h after incubation.

### 2.6. Albumin-Based Nanoparticles

Albumin is a main protein of plasma protein with a molecular weight of 66.5 kDa. High stability in pH (from 4 to 9) and heat (<60 °C), preferential uptake in tumor and inflamed tissue, biodegradability, low toxicity, immunogenicity and suitable blood circulation with a half-time of 19 days make albumins an ideal material as a drug delivery carrier [58]. In addition, albumin exhibits high binding capacity due to the multiple drug binding sites. Thus, not only noncovalent loading of drugs, but also covalent derivatization of albumin nanoparticles with drug targeting ligand is possible [98].

**Figure 1:**
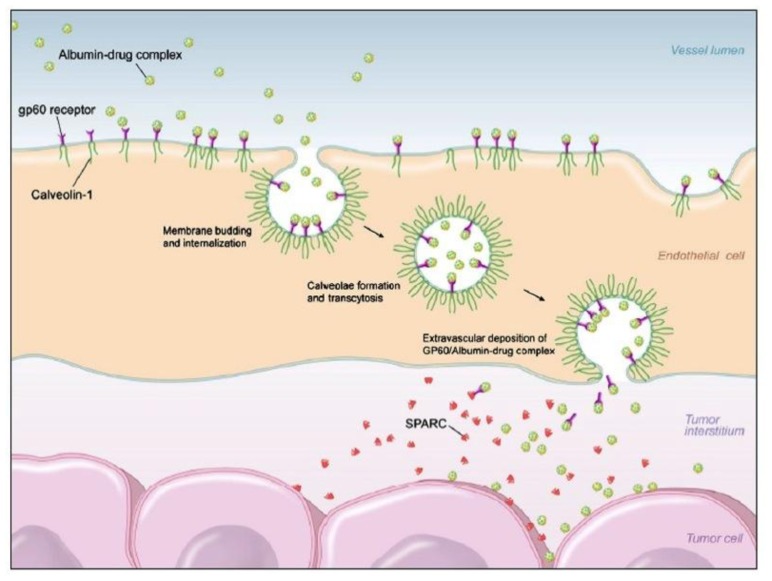
Uptake of albumin-paclitaxel nanoparticles is presumably mediated by the gp60 transcytosis pathway and subsequent binding to SPARC (Secreted Protein, Acidic and Richin Cysteine) in the tumor extracellular matrix. Reproduced with permission from Elsadek *et al.*, Journal of Controlled Release; published by Elsevier, 2012.

Human serum albumin (HSA) is one of the smallest and the most abundant proteins present in blood plasma, indicating many metabolic compounds and therapeutic drugs are transported by HSA. HSA can bind to albumin-binding proteins such as membrane-associated gp60 (albondin) and secreted protein, acidic and rich in cysteine (SPARC). Albondin receptor on the endothelial cells of tumor vassels allows transcytosis of albumin across continuous endothelium while overexpressed SPARC results in accumulation of albumin within the tumor interstitium (Figure 1) [99,100]. In consequence, albumin accumulates in malignant and inflamed tissues due to a leaky capillary combined with an absent or defective lymphatic drainage system. HSA has been known to be a suitable agent for gene therapy since it avoids undesired interaction with serum, which often occurs after intravenous injection of transfection complexes. In addition to blood derived albumin, recombinant human serum albumin (Recombumin) has also been developed and their safety, tolerability, pharmacokinetics and pharmacaodynamics to native HSA have been shown [101].

The preparation of uniformly sized albumin particles has been reported since 1970s. Coaservation [102], controlled desolvation [103,104], thermal gelation, emulsion formation [105] and self-assembly are main procedures that have been reported to prepare albumin-based nanoparticles. Coupling of low molecular weight drugs to exogenous or endogenous albumin, conjugation with bioactive proteins and encapsulation of drugs into albumin nanoparticles are main strategies for utilization of albumin as DDS carriers [58]. Albumin nanoparticles have been obtained by a continuous dropwise addition of ethanol to an aqueous solution of albumin with stirring due to the phase separation (coaservation) [106]. To stabilize the morphology of the nanoparticles, additional treatments such as crosslinking are often required. Thus, HSA-based nanoparticles were prepared by desolvation process followed by stabilization via crosslinking with glutaraldehyde as a crosslinker or heat denaturation [103]. Sulfhydryl groups were further introduced into HSA-based nanoparticles to increase the reactive sites for the covalent linkage of drugs to the particle surfaces. In this case, the ɛ-amino groups of lysine, the carboxyl groups of asparaginic and glutaminic acid, the carbonyl groups of the cross-linker glutaraldehyde and the sulfhydryl groups were the reactive sites on particle surfaces [107]. To eliminate the use of chemical crosslinkers such as glutaraldehyde to improve their biocompatibility, nanoparticles were prepared by complex coacervation technique using albumin and gum arabic (Acacia), a highly branched anionic polysaccharide via oppositely charged polyions interaction [102]. Loading of cancer drugs into albumin-based nanoparticles was achieved by coacervation of disulfide bond reduced BSA and thiolated alginate (alginate-cysteine conjugate) [108]. Cell uptake of nanoparticles occurred in both MCF-7 cells (human breast adenocarcinoma cells) and HeLa cells (human cervical cancer cells) and the presence of polysaccharide in the nanoparticle composition allowed a better interaction with cells. Nanoparticle albumin-bound (nab)-technology is a new technology for anti-cancer drug delivery system that has been developed by American Bioscience, Inc. Albumin particles with paclitaxel (nab-paclitaxel, 100–200 nm) have been approved in 2006 for use in patients with metastatic breast cancer due to their superior antitumor efficacy over paclitaxel [43]. The high efficacy of albumin particles over paclitaxel was due to the more effective intratumoral accumulation of drugs.

Core-shell nanoparticles were prepared from ovalbumin and lysozyme using thermal gelation method [109]. Thermal gelation of albumin occurred by heat-induced unfolding followed by protein-protein interactions including hydrogen bonding, electrostatic, hydrophobic interactions and disulfide-sulfydryl interchange reaction [110]. Ibuprofen encapsulated BSA-dextran nanoparticles (70 nm) were also obtained by a heat treatment. In this case, the conjugation of dextran to BSA stabilized the nanoparticles in aqueous solution.

Self-assembly of albumin to form nanoparticles can be achieved by the addition of lipophilic drugs and diminishment of primary amino groups on protein surfaces [111]. The disulfide bond breaking triggered unfolding, and assembly of HSA in a lipophilic drug-dependent technique to fabricate nanoparticles encapsulating hydrophobic molecules has been developed [112]. In this technique, HSA coassembled with lipophilic drugs, which acts as a bridge to form core–shell nanoparticles about 50–240 nm in size, and that the drugs bound to the subdomain IIA sites of HSA. These nanoparticles were stable in 5% glucose buffer and had tumor-targeting ability as demonstrated via *in vivo* fluorescent image. Paclitaxel-albumin nanoparticles carrying the homing peptides were also prepared by self-assembly. These nanoparticles were designed to target extravascular tumors, showing that nanoparticles can be effectively targeted into extravascular tumor tissue and that targeting can enhance the activity of a therapeutic nanoparticle [113].

### 2.7. Polypeptide Nanoparticles

A number of protein-mimicked polypeptide-based nanoparticles has been designed and synthesized to make the most of the unique features of proteins. These molecules have a well-defined composition, monodisperse molecular weight and potential biocompatibility [54,114,115]. The most often fabricated polypeptides are elastin-like polypeptides (ELP) that consist of alternating hydrophobic blocks and crosslinking domains [52,53,116–118]. ELP can be produced recombinantly and is composed of the repeating amino acid sequence of (Val-Pro-Gly-Xaa-Gly)*_m_*, where Xaa is the hydrophobic domain that facilitate both self-aggregation and elastomeric functions. Thus, ELP has a unique phase behavior, which promotes recombinant expression, protein purification and self-assembly of nanostructures. Considering that polymer composition, chain length and concentration, solvent composition, and solution temperature are the driving forces for the self-assembly of ELP, the synthesis of ELP via “recursive directional ligation” (RDL) is suitable to manipulate the precise architecture of ELP [52,118].

Nanoparticles were produced by self-assembly of ELP with a sequence of VPAVG and showed a sustained release of loaded dexamethasone phosphate for about 30 days [119]. The particles were stable either at room or body temperature, as they did not redissolve until a strong undercooling occurred. Well-designed ELP block copolymers are often produced to control their phase separation behavior, to add stimuli-responsivity and to introduce crosslinking domain into ELP. Temperature-triggered micelle assembly of ELP was achieved by the modulation of the local density of arginine (Arg) residues of diblock ELP. These micelles exhibited 8-fold increase in cellular uptake compared to the same ELP at a temperature where the polymer formed a soluble unimer. This off-on control of cellular uptake activity was due to the localization of cell penetrating Arg residues on the exterior of the nanoparticles [117]. Furthermore, pH sensitivity could be introduced to ELP micelles by using histidine-rich ELP block copolymers [116]. These micelles were dissociated at the low extracellular pH of solid tumors, indicating their application for tumor-specific drug targeting. ELP-based nanoparticles (~40 nm) were further formed from the diblock ELP decorated with the knob domain of adenovirus serotype 5 fibrous proteins for drug and gene delivery. Knob–ELP nanoparticles were internalized and localized to lysosomes of hepatocytes, which were mediated by knob-coxsachievirus and adenovirus receptor binding and endocytosis [53].

Similarly to ELP, silk-elastin-like protein (SELP) polymeric nanoparticles have unique physical and biological properties corresponded from both silk and elastin. Thus, they have been widely used in drug delivery and tissue engineering fields. They are usually formed by two steps; (1) formation of micellar-like particles with the silk blocks as the core structure by hydrogen bonding, (2) the hydrophobic interaction between elastin blocks above a specific transition temperature, which leads to the ordered association of SELPs molecules [120]. Other polypeptides are also produced recombinantly to form nanoparticles. Nanoparticles (100–200 nm) formed from cationic polyarginine and anionic hyaluronic acid is one of the examples [121]. Another example is the zwitterionic diblock copolymer consisting of poly(l-glutamic acid)-*b*-poly(l-lysine) (PGA-*b*-PLys). This block copolymer self-assembled into schizophrenic vesicles that can reversibly be produced in moderate acidic or basic aqueous solutions. The pH-dependent vesicle formation was due to the rod-like conformation of polypeptides in the hydrophobic area of the membrane [122].

## 3. Polysaccharide Nanoparticles

Polysaccharides are long carbohydrate molecules of repeated monosaccharide units joined together by glycosidic bonds. Chitosan, alginate, heparin, hyaluronic acid, pullulan and dextran are examples of polysaccharides [19,123–126]. They are naturally derived polymers with storage and structural functions, which are one of the main constituents in biological systems such as the glycocalyx and the extracellular matrices. Polysaccharides are highly stable, biocompatible and biodegradable. Thus, polysaccharides and their derivatives are commonly used for applications in food, biomedical and environmental fields [127,128]. Polysaccharides that have been used for preparation of nanoparticles are classified by their native charges; cationic (chitosan), anionic (alginate, heparin, hyaluronic acid) and nonionic (pullulan, dextran). Because of the presence of various derivable groups on molecular chains, polysaccharides can be easily modified both chemically and biochemically. Most natural polysaccharides have hydrophilic groups such as hydroxyl, carboxyl and amino groups, which also affect the polymer charges and could form noncovalent bioadhesion or react with functional molecules. Polysaccharide-based nanoparticles also can be categorized according to the mechanism of their formation; chemically crosslinked nanoparticles, physically crosslinked nanoparticles, polyion complex and self-assembled nanoparticles [19].

The most commonly used polysaccharide for nanoparticle fabrication is chitosan. Chitosan is a linear cationic heteropolymer of *N*-acetyl-*d*-glucosamine and d-glucosamine linked by beta-(1–4)glycosidic bonds, and is obtained by the partial deacetylation of naturally derived chitin. Chitosan is hydrophilic and soluble in acidic solution by protonation of the amine groups, and is degraded by enzymes such as lysozymes, some lipases and proteases. Chitosan increases cell membrane permeability, thus, can act as an absorption enhancer across intestinal epithelia prolonging the residence time of delivery systems at absorption sites, and has the ability to open the tight junctions of cell membranes [129]. Chitosan based delivery systems have been described for nasal, ocular, oral, parenteral and transdermal drug delivery. In particular, due to is mucoadhesive property, chitosan based gene delivery systems have been successfully applied to oral and nasal route gene therapy system, which will be discussed below [130].

Chitosan nanoparticles have also been used as carriers of growth factors such as epidermal growth factor and fibroblast growth factor, which could ultimately be impregnated into engineered tissue construct. Growth factors are essential in cellular signaling for migration, proliferation, differentiation and maturation [131].

Alginate is another example of polysaccharides that are often used for nanoparticle production. Alginate is a linear anionic polysaccharide composed of alternating blocks of 1,4-linked β-d-mannuronic acid (M) and α-l-guluronic acid (G) residues. Alginate has some advantages in its high mucoadhesiveness, aqueous solubility, and a tendency for gelation in proper condition, biocompatibility and non-toxicity [17,107,132]. Alginate can form network upon ionic inter- and intramolcular crosslinking with divalent ions. Calcium chloride is favorably utilized as a counter-ion for crosslinking nanogel formation. The number of studies involving alginate-based nanoparticles is increasing in delivery of therapeutic agents such as insulin, antitubercular and antifungal drugs [108,130,133–135]. Insulin-loaded nanoparticles were prepared from calcium crosslinking, alginate-chitosan or –alginate complexes with sufficient loading capacity of insulin [17,130,134,135]. Even delivery of genes loaded in alginate-based nanoparticles has been successful [133]. In this study, alginate-chitosan nanoparticles showed high transfection ability while maintaining biocompatibility and low toxicity.

### 3.1. Polysaccharide Nanoparticles by Crosslinking

Preparation of polysaccharide nanoparticles by crosslinking can be achieved by either ionic crosslinking (physical crosslinking) or covalent crosslinking (chemical crosslinking). Covalently crosslinked polysaccharide nanoparticles enable the network structure to be permanent since irreversible chemical links are formed unless biodegradable or stimuli-responsive crosslinkers are employed. The rigid network allows absorption of water and bioactive compounds without dissolution of the nanoparticles even when the pH drastically changes [136]. Chemical crosslinking of chitosan has been achieved by covalent bonds between polysaccharide main chains via crosslinkers such as non-natural glutaraldehyde [137] or low toxic di- and tricarboxylic acid (cuccinic acid, malic acid, tartaric acid and citric acid) [138]. 5-Fluorouracil loaded chitosan nanoparticles were produced by a water-in-oil emulsion method followed by glutaraldehyde crosslinking of the chitosan amino groups [139]. Cationic, anionic, ampholytic, or uncharged cross-linked nanoparticles (270–370 nm) were prepared by crosslinking chitosan using natural di- and trifunctional carboxylic acids as crosslinkers at different ratios [138]. Genipin, a natural biocompatible crosslinker isolated from the fruiets of Gardenia jasminoides Ellis, can be also used as crosslinkers of amino groups on chitosan backbones [140]. Covalent crosslinking was also demonstrated with other type of polysaccharides. An oil in water (o/w) emulsion polymer crosslinking method was employed for preparation of tamoxifen citrate (a non-steroidal antiestrogenic drug)-loaded guar gum nanoparticles crosslinked with glutaraldehyde. *In vivo* studies on female albino mice demonstrated maximum uptake of the drug by mammary tissue after 24 h administration with drug loaded guar gum nanoparticles, indicating their utilization for breast cancer treatments [141].

Physical crosslinking of polysaccharides is based on ionic interactions between charged polysaccharides and ionic crosslinkers. This method gives nanoparticles with reversibility and is considered biocompatible due to the lack of harsh preparation condition or toxic crosslinkers. Ionically-crosslinked nanoparticles are generally pH sensitive, which is a suitable feature for stimuli-sensitive controlled release. Tripolyphosphate (TPP), a non-toxic anionic molecule, has been widely used for the preparation of crosslinked chitosan nanoparticles, and a number of drugs have been encapsulated within these nanoparticles [142]. TPP crosslinked chitosan nanoparticles have been used for protein, oligonucleotides and plasmid DNA deliveries, due to their high physical stability and encapsulation efficiencies [143]. CS/TPP nanoparticles (300 nm) showed high encapsulation efficiencies both for plasmid DNA and dsDNA oligomers (20-mers), high physical stability and high gene expression levels in HEK 293 cells. Chitosan/TPP nanoparticles were also used for the improvement of the delivery of cyclosporine A to the ocular mucosa. Cyclosporine A was specifically concentrated in external ocular tissues (*i.e*., cornea and conjunctiva) during at least 48 h with maintaining negligible or undetectable CyA levels in inner ocular structures, blood and plasma [144]. Another examples of ionic crosslinking of polysaccharides are using of inorganic ions such as Fe(CN)_6_^4−^, Fe(CN)_6_^3−^ citrate and calcium ions as crosslinkers [145]. For example, carboxylic acids of alginate were crosslinked by calcium ions to form nanoparticles (80 nm), exhibiting high transfection rate of plasmid DNA into non-phagocytic cells via endocytosis pathway [135].

### 3.2. Polysaccharide Nanoparticles by Polyion-Complex

Polysaccharide nanoparticles are also prepared by direct electrostatic interactions of oppositely charged polysaccharides in solution. The stability of polyion-complex are determined by the degree of interaction between the polyelectrolytes, which is affected by the charge density and distribution, chemical environment such as pH of the solution, ionic strength, the temperature and the molecular weight of the polyelectrolytes and their flexibility. Chitosan is the most commonly used cationic polysaccharide for polyion-complex, whereas carboxymethyl cellulose (CMC) [146,147], dextran sulfate, carrageenan, heparin [124], hyaluronic acid [148], alginate and carboxymethyl pachyman are used as anionic polysaccharides [136,149]. Chitosan-CMC was subsequently coated with plasmid DNA for genetic immunization [146]. Both chitosan and a chitosan oligomer could complex CMC to form stable cationic nanoparticles for subsequent plasmid DNA coating. Chitosan/carrageenan/TPP nanoparticles (150–300 nm) were prepared by corporation of polyelectrolyte complexation and ionic gelation [149]. Plasmid DNA was loaded in chitosan-hyaluronic acid nanoparticles (110–230 nm) with high DNA association efficiency of 87%–99% (*w/w*) and transfection levels up to 25% GFP expressing HEK 293 cells [148]. Chitosan and growth factor binding heparin formed nanoparticles to enhance peripheral nerve regeneration resulting in the successful drug targeting in sciatic nerve [150]. Chitosan-alginate nanoparticles were prepared from dilute alginate sol by inducing a pre-gel with calcium counter ions, followed by polyelectrolyte complex coating with chitosan. Sufficient loading capacity of insulin in the nanoparticles showed their ability for application as an oral insulin delivery [130]. Chitosan-cyclodextrin nanoparticles were produced to encapsulate sulindac as a model drug, and their continuous release of the drug throughout 24 h was detected [151].

Not only polysaccharides, anionic polypeptides are also used for polyelectrolyte complexation with chitosan. PH-sensitive polyion complexes (average particle size <200 nm) were formed from trimethyl chitosan and α-galactosidase A through self-assembly. These nanoparticles were able to release the enzyme at acidic pH and were efficiently internalized by human endothelial cells and mostly accumulated in lysosomal compartments [152]. γ-Poly(glutamic acid) (PGA) was combined with chitosan to form nanoparticles for transdermal delivery of DNA [125]. These nanoparticles enhanced skin penetration and gene expression in comparison to particles solely comprised for chitosan and DNA, due to the high penetration ability of PGA into the skin barrier. pH-sensitivity was added to chitosan-PGA nanoparticles by mixing PGA solution with chitosan solution in the presence of MgSO_4_ and sodium tripolyphosphate [153]. Both *in vitro* and *in vivo* studies showed that these nanoparticles could effectively infiltrate into the mucosal cell membrane and enhance hypoglycemic action by oral administration of insulin-loaded nanoparticles.

Chitosan-DNA complex and chitosan-plasmid DNA complex are other examples of nanoparticles derived from chitosan-based polyion-complex. The chitosan-plasmid DNA nanoparticles were formulated using complex coacervation and solvent evaporation technique, showing that the release of plasmid DNA from complexes was with a range of 24–96 h depending on the preparation process [154].

### 3.3. Polysaccharide Nanoparticles by Self-Assembly

Introduction of hydrophobic segments into hydrophilic polysaccharide backbones enables to form self-assembled structures such as micelles, particles and hydrogels [155,156]. Deoxycholic acid, cholesterol, carboxylic acids and hydrophobic polymers are examples of such hydrophobic segments [155,157,158]. By manipulating the introduction condition such as polysaccharide/hydrophobic segments molar ratios and the lengths of both polysaccharides and hydrophobic segments, nanoparticles are formed to minimize interfacial free energy. Introduction of hydrophobic segments into polysaccharides is achieved by grafting hydrophobic groups from hydroxyl, amino or carboxyl groups of the polysaccharide main chains. These chemically modified amphiphilic macromolecules can self-associate in aqueous solution by intra- and/or inter-molecular hydrophobic interaction, which can form nanoparticles [155]. Water insoluble drugs are solubilized and encapsulated within the hydrophobic core and become soluble in water due to the hydrophilic shell. The drugs are then released from the inner core of the nanoparticles via outer stimuli changes such as pH, temperature and ionic strength.

Recently, there have been a number of studies on the syntheses of polysaccharide-based self-aggregated nanoparticles for drug delivery system [159]. Chitosan has been chemically modified by grafting hydrophobic groups from amino groups of the main chains, where the proportion of the amine groups depends on the acetylation degree of the polymer. Grafting of the hydrophobic groups into amino groups of chitosan was achieved via acylation by acyl chloride or acid anhydride, coupling with deoxycholic acid using a coupling agent [160] or reaction with cyclic anhydrides [161]. Doxorubicin, paclitaxel, ibuprofen and the anphiphilic adriamycin have been loaded in chitosan based nanoparticles. Plasmid DNA was introduced into nanoparticles (160 nm) aggregated from deoxycholic acid-grafted chitosan, and their efficient transfection of COS-1 cells was detected [160].

Dextran-based self-assembled nanoparticles are another candidate for drug/gene delivery carriers. Dextran was chemically modified by grafting bile acids, natural product consisting of a facially amphiphilic steroid nucleus with a hydrophobic β-side and a hydrophilic α-side, or lauryl chains [162]. Mannose groups were also conjugated on the azido-bearing dextran nanoparticles via click chemistry, exhibiting enhanced antigen presentation in the context of major histocompatibility complex class I molecules due to the internalization and activation of antigen presenting cells [163]. Self-assembly assisted graft copolymerization of acrylic acid from dextran under the presence of crosslinker produced pH-sensitive nanoparticles (40–140 nm) [164,165]. Dextran could form interpolymer complexes with poly(acrylic acid) (PAA) in acid medium owing to hydrogen bond interaction of carboxyl groups and proton-acceptors in glucose units. Thus, the hydrophobic driving force for the formation of nanoparticles should be attributed to the complexation of PAA grafts and dextran segments. Similar nanoparticles were formed from hydroxypropyl and hydroxyethyl celluloses [165]. Comb-shaped copolymers composed of dextran backbones and cationic poly((2-dimethyl amino)ethyl methacrylate) side chains were subsequently prepared via atom transfer radical polymerization for nonviral gene delivery. Plasmid DNA were loaded into complex nanoparticles (100–150 nm), which had high gene transfection yield, efficient gene delivery ability in different cancer cell lines, especially in MCF-7cells [166]. Similarly to chitosan and dextran, hyaluronic acid (HA) was chemically modified with the 5β-cholanic acid to form self-assembled nanoparticle (200–400 nm) that combine both passive tumor targeting based on the EPR effect and a more specific or active targeting exploiting the affinity of HA towards CD44 [167]. Amphiphilic block copolymers were also synthesized to form nanoparticles via self-assembly. Doxorubicin loaded nanoparticles based on poly(γ-benzyl l-glutamate)-*block*-hyaluronan were produced by self-assembly. These particles could be used as a self-targeting drug delivery cargo in over-expressed CD44 glycoprotein cells of breast cancer [168].

Nonionic pullulan is also modified by hydrophobic molecules such as cholesterol and cancer drugs. The cholesterol-bearing pullulans with different molecular weights and degree of substitution have been synthesized to form self-assembled nanoparticles [157]. Monodisperse self-aggregates (20–30 nm) have been formed from these grafted pullulans due to the association of cholesteryl moieties. Recent study demonstrated paclitaxel-incorporated nanoparticles prepared from pullulan hydrophobically modified by acetic anhydride to evaluate their antitumor activity *in vitro* and *in vivo* [169]. These nanoparticles showed lower antitumor activity *in vitro* against HCT116 human colon carcinoma cells, and reduction of tumor growth *in vivo* using HCT116 human colon carcinoma-bearing mice.

Starch-based nanoparticles have also been reported by several groups. Starch is the second most abundant biomass material in nature, found in plant roots, stalks, crop seeds. It consists of two main structural components, the amylose and amylopection [170,171]. Propyl-starch nanoparticles were reported to show the increase in the solubility of the polymer in low hazardous organic solvents and high encapsulation efficiency for model drugs [172]. Effective controlled release of doxorubicin was shown from drug-conjugated dialdehyde starch nanoparticles [173]. Docetaxel, an anti-cancer agent, was loaded in nanoparticles prepared from a hydrophobic propyl starch with a controlled degree of substitution via the solvent emulsification/diffusion technique. It was confirmed that nanoparticle enhanced internalization by the cancerous cells (Caco-2 and NHDF-p cells) and their peri-nuclear localization was detected [174].

Heparin, a negatively-charged polysaccharide that used as an anticoagulant, is often applied for the preparation of self-assembled nanoparticles. A self-assembled nanoparticulate system (140–190 nm) composed of a folate-conjugated heparin-poly(β-benzyl-l-aspartate) (HP) amphiphilic copolymer was proposed for targeted delivery of the antineoplastic drug paclitaxel. The presence of folate not only enhanced intracellular uptake via endocytosis, but also these nanoparticles exhibited a great cytotoxicity in KB cells [175]. Deoxycholic acid bearing heparin amphiphilic conjugates (120–200 nm) with different degrees of substitution were also synthesized [158].

## 4. Summary

Recent progresses in synthesis of biopolymer-based nanoparticles and their application as drug/gene carriers are introduced in this review. Biopolymers possess several favorable characteristics in comparison to synthetic polymers used in drug/gene delivery, such as biocompatibility, biodegradability and abundant renewable sources. Considering that size and distribution of particles are critical parameters to target specific organs and tissues, proteins are suitable materials for drug/gene carriers due to their precise molecular sizes derived from their secondary structure. Moreover, the features of proteins that tend to form self-assembled α-helix, β-sheet or random structures enable them to easily form unique nanostructures such as nanoparticles, nanofibers and nanosheets. To produce protein-based nanoparticles with ideal size, morphology and stability, several methods have been demonstrated, including crosslinking using crosslinkers, emulsion formation, coacervation and precipitation. Polysaccharides also offer a suitable platform to produce nanoparticles due to their biodegradability, biocompatibility and ease in molecular modification both covalently and ionically. Formation of nanoparticles from polysaccharides is achieved by ionic or covalent crosslinking, ion-complex and self-assembly after grafting of hydrophobic segments to the polymer backbone. In this regard, the choice of the most suitable technique for nanoparticle production depends on the nature of the materials such as charges and polymer chain lengths. Introduction of ligands onto the surface of the nanoparticles to target specific sites will further enable biopolymer nanoparticles to be used more often in clinical application. Not only protein- or polysaccharide-based nanoparticles, but also nanoparticles based on biopolymer-synthetic polymer conjugates have been also paid much attention. Such multi-functional biopolymer-based and biopolymer-synthetic polymer conjugated nanoparticles will become candidates of the precisely targeted delivery of drugs and genes in many therapeutic fields.
